# The CLE33 peptide represses phloem differentiation via autocrine and paracrine signaling in Arabidopsis

**DOI:** 10.1038/s42003-023-04972-2

**Published:** 2023-06-06

**Authors:** Samy Carbonnel, Salves Cornelis, Ora Hazak

**Affiliations:** Department of Biology, University of Fribourg, Chemin du Musee 10, 1700 Fribourg, Switzerland

**Keywords:** Plant development, Plant signalling

## Abstract

Plant meristems require a constant supply of photoassimilates and hormones to the dividing meristematic cells. In the growing root, such supply is delivered by protophloem sieve elements. Due to its preeminent function for the root apical meristem, protophloem is the first tissue to differentiate. This process is regulated by a genetic circuit involving in one side the positive regulators *DOF* transcription factors, *OCTOPUS* (*OPS*) and *BREVIX RADIX* (*BRX*), and in the other side the negative regulators *CLAVATA3/EMBRYO SURROUNDING REGION RELATED* (*CLE*) peptides and their cognate receptors *BARELY ANY MERISTEM* (*BAM*) receptor-like kinases. *brx* and *ops* mutants harbor a discontinuous protophloem that can be fully rescued by mutation in *BAM3*, but is only partially rescued when all three known phloem-specific *CLE* genes, *CLE25/26/45* are simultaneously mutated. Here we identify a *CLE* gene closely related to *CLE45*, named *CLE33*. We show that double mutant *cle33cle45* fully suppresses *brx* and *ops* protophloem phenotype. *CLE33* orthologs are found in basal angiosperms, monocots, and eudicots, and the gene duplication which gave rise to *CLE45* in Arabidopsis and other *Brassicaceae* appears to be a recent event. We thus discovered previously unidentified Arabidopsis *CLE* gene that is an essential player in protophloem formation.

## Abstract (web summary)

A previously unidentified CLE peptide, CLE33, is discovered and demonstrated to function redundantly with CLE45 to repress protophloem differentiation via the BAM3 receptor.

## Introduction

In vascular plants, phloem tissues transport sugars and signaling molecules to sink organs for growth and storage^1,2^. In the growing Arabidopsis root, the root apical meristem is a large sink and two phloem poles, containing functional sieve elements, unload the phloem sap in the meristematic region^3^. Each pole consists of a protophloem sieve element, flanked by two phloem pole pericycle cells from the outside, and a metaphloem sieve element from the inside, and two companion cells adjacent to these sieve element cells. This complex structure acts as a functional unit^4^. Remarkably, the delivery of sugars and hormones to the root meristem is uniquely mediated by protophloem sieve elements, while metaphloem sieve elements remain undifferentiated in this region of the root^5^.

The process of protophloem sieve element formation requires radical cellular modifications, including cell wall reinforcement, enucleation, and sieve plate formation^6,7^. The genetic control of protophloem development in Arabidopsis has been studied intensively in the last decade^4,6,8–11^, including precise transcriptomics analysis of the whole phloem pole^4^ and developing sieve elements^6^. Protophloem differentiation is controlled by multiple regulators, such as hormone gradients, DOF transcription factors, and SMXL transcriptional repressors^9,12^. In addition, two membrane-localized proteins, BREVIS RADIX (BRX) and OCTOPUS (OPS) act as positive regulators of protophloem development^8,10^. *brx* and *ops* loss-of-function mutants display a discontinuous protophloem, characterized by so-called gap cells that fail to differentiate. The interrupted protophloem continuity results in a reduced phloem sap delivery to the root apical meristem and limited root growth^8,10^. *BARELY ANY MERISTEM 3* (*BAM3*) was identified in a *brx* suppressor screen with *bam3* rescuing both root growth and gap cell phenotypes of *brx* and *ops*^10^. *BAM3* encodes for a leucine-rich repeat receptor kinase (LRR-RLK), a cognate receptor of the CLAVATA3/EMBRYO SURROUNDING REGION 45 (CLE45) peptide^13^. While a higher-order mutant of phloem-expressed *CLE* genes (*CLE25/26/45*) have been recently shown to partially rescue the *brx* and *ops* gap cell phenotype, this rescue is not to the level of the full rescue achieved in *bam3*^14^. This phenotypic discrepancy between the receptor and its ligands suggests that additional endogenous CLE peptides, BAM3 ligands, remain to be identified.

CLE peptides are produced from a roughly 100 amino acids pre-propeptide that possess a signal peptide in its N-terminus for apoplastic release, a central variable region, and a conserved 12–13 residues CLE domain in its C-terminus, which is cleaved off to release the mature CLE peptide. Given their small gene size and high sequence variability outside the CLE domain, the genome annotation of *CLE* genes can be challenging. In Arabidopsis, their discoveries came initially from a mutant screen^15,16^, following the cloning of *CLV3*^17^. The first release of the Arabidopsis genome^18^ allowed the identification of additional members of the *CLE* family in several iterations^19–23^. Up to date, 32 *CLE* genes are annotated encoding for 27 unique peptides. We recently explored the *CLE* gene family in tomato, and found 37 new *CLE* genes^24^, sparking the question whether additional *CLE* genes remain to be discovered in Arabidopsis.

Here we present the identification of the Arabidopsis *CLE33* gene, which was missed in previous genome analyses. We show that this gene is expressed in the developing root phloem and encodes for an active peptide which acts redundantly with CLE45 via BAM3 receptor in inhibiting protophloem differentiation in the protophloem cell lineage and neighboring cells.

## Results

### *CLE33* is a novel Arabidopsis peptide gene

**Fig. 1:**
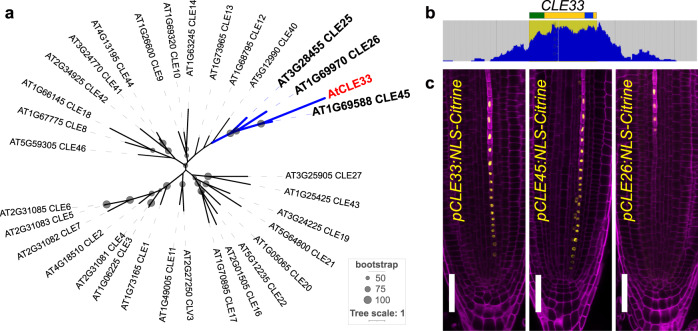
*CLE33* is a novel phloem-expressed peptide gene. **a** Phylogenetic tree of *Arabidopsis thaliana* full-length CLE pre-propeptides. The blue line indicates the cluster expressed in the root phloem tissue. **b** Araport11 RNA-seq-based evidence of transcription from mapping coverage of the light-grown seedlings visualized with JBrowse. The relative position of the predicted *CLE33* coding sequence is indicated schematically: signal peptide in green, CLE domain in blue. **c** Transcriptional fusions showing the promoter activity of *CLE33*, *CLE45* and *CLE26* in the root protophloem tissue. Scale bar = 50 μm.

To search for additional Arabidopsis *CLE* genes, we performed a non-stringent t-BLAST-n analysis using the 32 previously known full-length CLE proteins from *A. thaliana* as queries. We found one sequence in an unannotated region of chromosome 1 that shows all the characteristics of a *CLE* gene, including the presence of a functional signal peptide. In other *Brassicaceae* species, this genetic locus is conserved and is annotated as a *CLE45-like* homolog (Supplementary Fig. 1a). In *A. thaliana*, this locus is transcribed, confirming expression of the gene, which we named *CLE33* (Fig. 1b and Supplementary Fig. 1b). Based on its full-length protein sequence, we determined that *CLE33* is the closest homolog of the well-characterized phloem-expressed *CLE45* (Fig. 1a)^10,11,13^.

To get insights about the expression pattern of *CLE33*, we cloned its promoter region to drive a *GUS* reporter (Supplementary Fig. 1c). *CLE33* expression was specifically associated with vasculature in the root meristem, rosette junction and leaves, cotyledons, and inflorescence stem. To precisely determine the expressed tissue, we use the same promoter and two additional phloem-specific *CLE*s (*CLE26* and *CLE45*) to drive the expression of nuclear fluorescent protein NLS-Citrine (Fig. 1c and Supplementary Fig. 1d). In the root meristem, all three are specifically expressed in protophloem tissue, but with slight differences in the expression onset. *CLE45* expression can be observed early in the sieve element precursor cells, immediately after the first periclinal cell division of the sieve element procambium precursor. *CLE33* expression starts a few cells later, in the developing protophloem sieve element cells, largely overlapping with the expression domain of *CLE45*. *CLE26* is active in the later stages of sieve element differentiation, when the cell wall thickening has happened, in accordance with the previously published reporters^25,26^.

### *CLE33* encodes for a root-active peptide perceived by BAM3

**Fig. 2:**
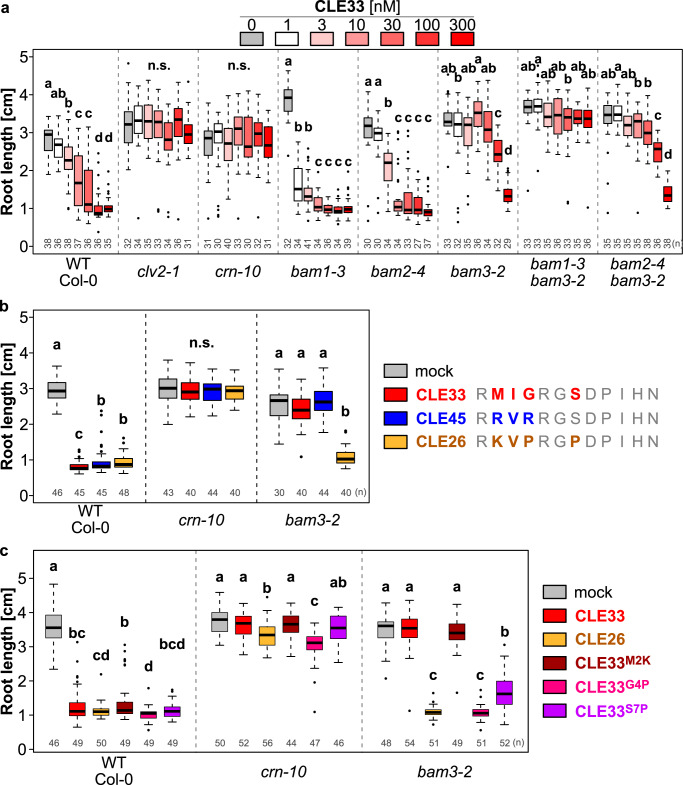
CLE33 is a root-active peptide inhibiting root growth in a BAM3-dependent manner. **a** Dose-dependent root growth inhibition by CLE33p in the wild type and CLE receptor mutants. **b** Root growth inhibition with synthetic CLE33 (30 nM), CLE45 (30 nM), CLE26 (3 nM) peptides. **c** Root growth inhibition of CLE33 peptide variants CLE33^M2K^, CLE33^G4P^, CLE33^S7P^ (30 nM). **a**–**c** Letters indicate different statistical groups (ANOVA, post hoc Tukey test).

Most CLE peptides induce root growth arrest when overexpressed or when added to the growth media at nanomolar concentrations and are therefore called root-active^13,26^. To ascertain whether CLE33 peptide could also inhibit primary root growth and which receptors are involved in perceiving this peptide in the root meristem, we applied CLE33 peptide at increasing concentration from 0 to 300 nM in the growth media and assessed the root growth inhibition effect in the wild-type and loss-of-function CLE receptor mutants (Fig. 2a). In the wild type, the root growth was inhibited with 3 nM of CLE33, becoming more pronounced with higher concentrations. As for other known root-active CLEs, this response was fully *CLV2*/*CORYNE*-dependent^13^, even at the highest concentrations. In contrast, *bam* mutants exhibited a variety of responses. *bam2-4* showed similar sensitivity to the wild type, whereas *bam1-3* was hypersensitive to CLE33p. This hypersensitivity of around 3-fold was also observed with CLE26 and CLE45 peptides (Supplementary Fig. 2a, b), and to CLE33 peptide in the independent *bam1-4* allele (Supplementary Fig. 2d). On the other hand, *bam3* mutants were insensitive up to 100 nM of CLE33 peptide, and only showed a response at very high concentration of 300 nM (Fig. 2a and Supplementary Fig. 2c). Only in combination of *BAM1* loss-of-function, full insensitiveness was achieved, suggesting that *BAM1* plays a role in mediating root growth responses to synthetic CLE33 peptides at high concentration. To further test this finding under closer physiological conditions, we used the XVE-estradiol inducible system to ectopically express *CLE33* (Supplementary Fig. 3). While the wild type strongly reacted to the ectopic induction of *CLE33*, *bam3-2* lines remained insensitive even at high estradiol concentration. These results suggest that synthetic and endogenous CLE33 peptides require *BAM3* for root growth inhibition effect.

BAM3 was shown to be the cognate receptor of CLE45 peptide^13^, and *bam3* mutants present a similar insensitivity to CLE33 and CLE45 peptides (Fig. 2b and Supplementary Fig. 2a). This unique BAM3-dependency is very specific to CLE33 and CLE45 peptides, but not to the closely related CLE26 peptide (Supplementary Fig. 2b). By comparing the amino acid sequences of these three peptides, we noticed that the conserved prolines at the positions 4 and 7 are replaced in CLE33 by glycine and serine, and in CLE45 by arginine and serine (Fig. 2b). To further investigate the importance of these amino acids for receptor specificity, we created and tested variants of CLE26 and CLE33 by swapping residues between these two peptides. First, we observed that all the peptide variants (CLE26^P4G^, CLE26^P7S^, CLE26^P4G/P7S^, CLE33^M2K^, CLE33^G4P^, CLE33^S7P^) had a strong root growth inhibition effect in wild type but largely no effect in *crn-10* mutant, which implies that the amino acids substitutions did not affect their ability to suppress root growth (Fig. 2c and Supplementary Fig. 4). However, the BAM3-dependency was strongly relying on the residues at positions 4 and 7. The presence of a proline at position 4 or 7 in CLE33 led to a BAM3 independent response (Fig. 2c), and inversely both prolines needed to be replaced in CLE26 to not inhibit root growth in the *bam3* mutant (Supplementary Fig. 4). Taken together, these results suggest that the absence of prolines in position 4 and 7 determine the BAM3 perception specificity of the CLE33 and CLE45 peptides.

### CLE33 and CLE45 act in concert in protophloem differentiation

It has been previously shown that root protophloem development is suppressed by root-active CLE peptides added to the growth media^13^. To test the effect of CLE33 on protophloem identity, we evaluated the expression of the protophloem marker *pCVP2:NLS-3xVenus* upon overnight treatment of CLE33 peptide (Supplementary Fig. 5). The inhibitory effect of CLE33 on protophloem identity was similar to the effect of CLE26 and CLE45, suggesting that CLE33 acts as a negative regulator of protophloem differentiation.

**Fig. 3:**
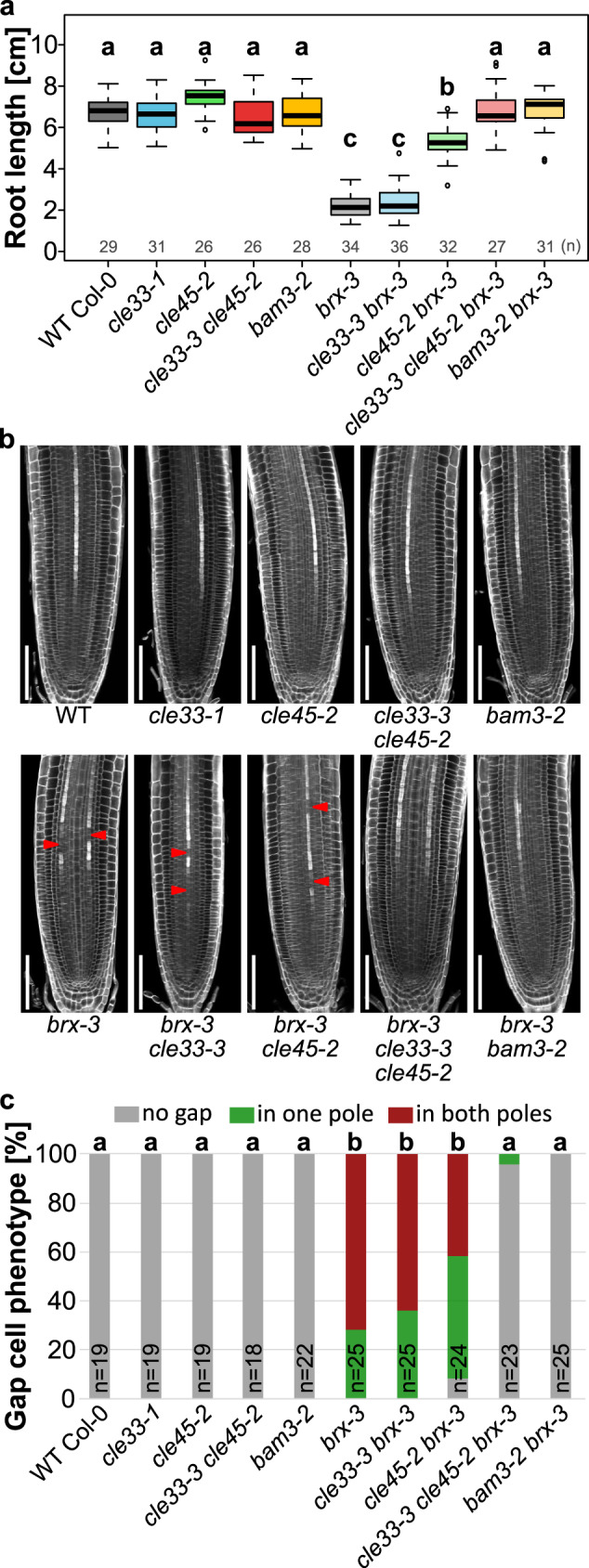
Functional redundancy of *CLE33* and *CLE45* in *BRX*-mediated regulation of protophloem development. **a** Root length at 10 days post germination. Letters indicate different statistical group (ANOVA, post hoc Tukey test). **b** Representative confocal images of calcofluor white stained roots. Red arrows indicate protophloem gap cells. Scale bars correspond to 100 μm. **c** Gap cell frequency quantification. Letters indicate statistical groups (*χ*^2^ test with Benjamini–Hochberg correction).

To understand the biological function of *CLE33 in planta* and its genetic relationship with *CLE45*, we created CRISPR-Cas9 mediated knock-out mutants in wild type and *cle45-2* backgrounds (Supplementary Fig. 6). Alike *bam3*¸ the single *cle33* and double *cle33cle45* mutants do not show any reproducible root growth macro-phenotype in our conditions (Fig. 3 and Supplementary Fig. 7). In accordance to previous reports, *BAM3* loss-of-function mutants can fully rescue the discontinuous protophloem differentiation of *brx* and *ops*^8,10^, which is not the case for *cle45*^14^. We asked whether this phenotypic discrepancy could be attributed to the presence of the remaining *CLE33* in protophloem tissue. To explore this further, we crossed the double mutant *cle33-3 cle45-2* with *brx-3* and *ops-*2. Mutation in *CLE33* alone had no or very little impact on rescuing root growth and protophloem continuity of *brx-3* and *ops-2* (Fig. 3 and Supplementary Fig. 7). We could reproduce a previously published result, where *cle45* partially suppressed the root growth phenotype but failed to significantly reduce the protophloem gap cell frequency^14^. The combination of *cle33* and *cle45* could fully rescue the root growth and protophloem discontinuity phenotypes, strongly suggesting that *CLE33* and *CLE45* act redundantly in this genetic circuit (Fig. 3 and Supplementary Fig. 7). These results taken together support a negative role for *CLE33* in protophloem differentiation.

The protophloem development failure caused by *BRX* or *OPS* loss-of-function could be explained by high dosages of the receptor *BAM3* or phloem-specific *CLE* ligands. To test this possibility, we quantified transcript accumulation in *brx* and *ops* root tissues. We found, that on the transcriptional level *BAM3* is not up-regulated and *CLE25*, *CLE26* and *CLE45* even showed significant reduction in their expression (Supplementary Fig. 8a), suggesting that the increased BAM3/CLE33/45- mediated signaling in *brx* and *ops* mutant is caused by another mechanism.

**Fig. 4:**
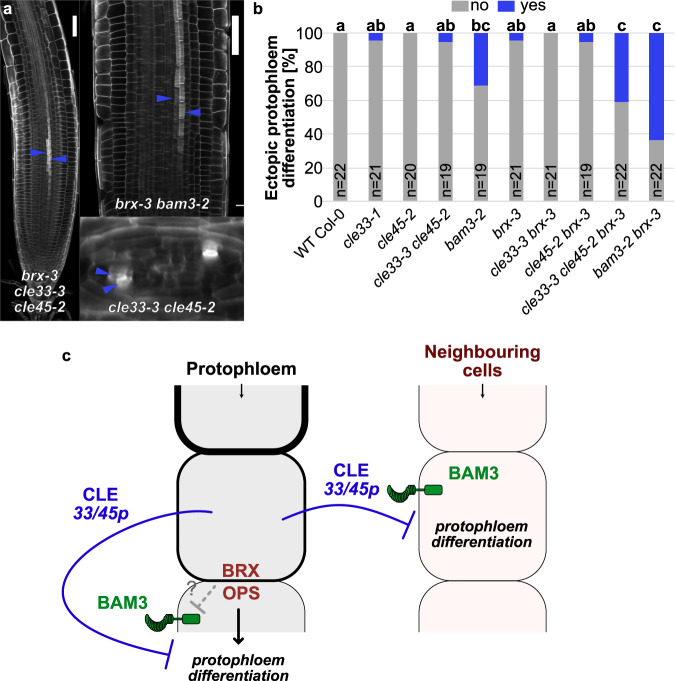
Protophloem-expressed CLE33 and CLE45 peptides inhibit ectopic protophloem differentiation in neighboring cells. **a** Confocal images of calcofluor white stained roots. Blue arrows indicate cell files showing a cell wall reinforcement, sign that they are undergoing a protophloem differentiation process. Scale bars correspond to 50 μm. **b** Frequency of ectopic protophloem differentiation in roots. Letters indicate statistical groups (*χ*^2^ test with Benjamini–Hochberg correction). **c** Schematic model of autocrine and paracrine signaling mediated by the CLE33/45 peptides via the BAM3 receptor.

While analyzing the protophloem continuity, we came across an additional phenotype of ectopic sieve element-like cells originated from the neighboring cells adjacent to the main protophloem sieve element cell file. These cell files show characteristics of developing sieve elements, including thickening of the cell wall (Fig. 4a). A related phenotype was recently shown in the multiple loss-of-function mutants in phloem *CLEs, BAM* receptor, and *CIK* co-receptor mutants, which demonstrated that *CLE* signaling restricts neighboring cells to differentiate into sieve elements^9^. This previous analysis was performed at a later stage in phloem development when both protophloem sieve elements and metaphloem sieve elements have differentiated and possess thick cell walls^9^. In our analyses, we focused on the meristematic zone. We quantified the frequency of the ectopic protophloem differentiation events in our mutants. We found an increased ectopic differentiation in the absence of *BAM3* and in the triple mutants *cle33 cle45 brx* and *cle33 cle45 ops*, but not in the double mutant combinations (Fig. 4b and Supplementary Fig. 8b). This difference could be due to a weaker phloem identity in the absence of *brx* and *ops*, and/or reduced levels of *CLE25/CLE26* in these backgrounds (Supplementary Fig. 8a) which are known to repress ectopic sieve element formation^9^. Nevertheless, the results of our analysis show that *CLE33* and *CLE45* act redundantly in inhibiting the formation of the ectopic protophloem sieve elements (Fig. 4c and Supplementary Fig. 7).

It has been recently demonstrated that *CLE25*, *CLE26*, and *CLE45* have DOF-transcription factor binding sites in their promoter region and are induced by *DOF2.2*^9^. Our analysis of the *CLE33* promoter region revealed similar binding sites for *DOF2.2*, *DOF2.4/PEAR1*, *DOF3.2*, *OBP3*, *DOF5.1/PEAR2*, and *DOF5.6* (Supplementary Fig. 8c). This result suggests that *CLE33* can be an essential player in DOF-CLE genetic circuit.

### Evolutionary origin of *CLE33* and *CLE45*

The two peptide-encoding genes, *CLE33* and *CLE45* appear to be functionally redundant (Figs. 3 and 4) and share a high sequence and expression pattern similarity (Fig. 1). Therefore, they could be the result of a gene duplication event. To learn about their evolutionary origin, we performed a phylogenetic analysis across diverse vascular plant species. We identified *CLE33* orthologs in the genomes of basal angiosperms, monocots, and eudicots, but not of gymnosperms, suggesting that it emerged ~200 million years ago (Supplementary Fig. 9a). Interestingly, clear *CLE45* orthologs were only found in *Brassicales* species. A multi-sequence alignment revealed that *Brassicales* CLE45 have an extended variable region with no homology to other CLE33/45-like proteins in the N-terminal part immediately after the signal peptide (Supplementary Fig. 9b). However, when analyzing specifically the CLE domain, we observed that *CLE45* is more similar to ancestral *CLE33* orthologs (Supplementary Fig. 9c). One possibility for such occurrence is that following a duplication in early *Brassicales*, one copy quickly drifted genetically in its variable region giving rise to the current *CLE45*. In such a scenario, *Brassicales CLE33* maintained most of the ancestral sequence, except for the residues 2/3/4 in its CLE domain. Strikingly, all the orthologs of both *CLE33* and *CLE45* do not have any prolines at positions 4 and 7, which we found to be consequential for receptor perception specificity in Arabidopsis (Fig. 2).

Furthermore, we investigated the evolutionary origin of *BAM3*, the gene encoding for the receptor perceiving these two ligands. We found orthologs present up to the gymnosperms (Supplementary Fig. 10). An early duplication in the angiosperms produced two copies: *BAM3* and *BAM4*. While *BAM3* is found in all angiosperm groups, *BAM4* is peculiarly absent from the genomes of *Brassicaceae* species. The loss of *BAM4* coincides with the emergence of *CLE45* in *Brassicales*. Yet, *BAM4* ligands and their functions remain unknown. Our findings open new directions for further exploration into these receptor-ligand pairs and their potential conserved function throughout plant evolution.

## Discussion

In recent years, many additional peptide ligand-encoding genes have been discovered in Arabidopsis and other plant species^24,27–31^. These belong to previously unknown families, such as the stress-induced *CTNIP*s/*SCREW*s peptides^27,28^, or well-characterized gene families, such as *CIF/TWS1*^31^ and *CLE* in our study. It seems that plant genome annotations, including Arabidopsis, still miss many of such peptide-encoding genes. However, advanced methods like ribosome profiling combined with transcriptomics and peptidomics^32,33^ have shown a great success in identifying a few thousands of such genes in *Medicago*^32^ and maize^33^. In addition, the genome-wide analyses using hidden Markov models^34^ led to the identification of new peptides^24,35^. With the aid of such advanced methods, we will be soon able to obtain a full repertoire of plant signaling peptides regulating a myriad of developmental and adaptive responses.

In our study, we performed a non-stringent t-BLAST-n search for *CLE* genes, which allowed us to identify the previously non-annotated *CLE33*. Prolines at positions 4 and 7 are among the most conserved amino acids in CLE peptides, and their absence in CLE33/45 makes them exceptions and partially explains why these genes were identified later^22^. From a structural point of view, the proline residue confers unique conformational changes because of its irregular geometry^36^. The side chain of the proline is connected to the peptide backbone twice, creating its own secondary structure. Little is known about the contribution of prolines to the interactions with the CLE receptor and co-receptor binding surfaces. However, it was shown that in mature CLE peptides, prolines 4 and 7 undergo hydroxylation and either or both undergo glycosylation with 3, 4, or 6 residues of arabinose^37^. The hydroxyproline in position 4 in CLE40 peptide was shown to prevent the miscleavage of the precursor peptide^38^. At the same time, absence or presence of hydroxylation in the mature CLE40 peptide and substitution of the proline in position 4 to alanine (P4A), did not affect the bioactivity of the peptide in root growth assays^38^. Furthermore, replacing hydroxyprolines in positions 4 and 7 by prolines in CLE9 did not affect binding affinity to BAM1 in vitro^39^, which could have suggested that proline 4 itself and proline hydroxylation are not essential for peptide–receptor interaction^38,39^. However, our bioactivity assays with modified CLE26 and CLE33 peptides indicate that prolines at positions 4 and 7 are determinants for receptor specificity. It has been demonstrated that in BAM3, the Q^226^Y^228^Y^231^ residues are crucial for CLE45 binding^13^, but how the absence of prolines 4 and 7 contribute to the specific binding with BAM3 needs further clarification.

The emergence of highly specialized sugar-conducting sieve elements was a crucial step in the evolution of land plants. This invention facilitated the separation of photosynthetic organs that need to compete for light and grow towards the sun, and water-absorbing organs that grow deep into the soil. Primitive sieve element files can be found in brown algae species and mosses^40^, often showing only partial protoplast degradation and enucleation. All non-flowering vascular plants, including gymnosperms, possess sieve cells, which have sieve areas and not sieve plates on both end and side walls^41^. In gymnosperms, the conducting sieve cells lack adjacent companion cells and are shaped like elongated spindle-like structures that are axially and laterally connected by sieve areas with narrow sieve pores, which leads to higher flow resistance and slower transport rates. Angiosperms develop highly efficient isolated phloem sieve elements with sieve plates on the end walls, which facilitate the fast transport of photoassimilates^41,42^. Here we identified a new molecular player in root protophloem formation, a small signaling peptide gene *CLE33*. In the Arabidopsis root, phloem-specific DOF transcription factors induce a set of positive regulators of phloem development, and *CLE*s, which downregulate DOF expression in a feedback loop^9,12^. With DOF binding sites in its upstream regulatory sequence, *CLE33* is likely involved in this genetic circuit. We demonstrated that *CLE33* acts in concert with *CLE45* to counteract sieve element differentiation mediated by *BRX*/*OPS*. Such a mechanism can potentially have a role in defining the right timing for differentiation of sieve element depending on endogenous cues and environmental conditions. Furthermore, we show that *CLE33* acts redundantly with *CLE45* to repress the protophloem neighboring cells from differentiating into sieve elements. These results suggest that *CLE33/45* act both as autocrine and paracrine signals, whereas the two other phloem *CLE* genes (*CLE25* and *CLE26*) have a limited role as autocrine signals in the *BRX/OPS-BAM3* module regulating protophloem differentiation. Another player in this pathway, *RECEPTOR PROTEIN LIKE KINASE 2* (*RPK2*) was recently shown to be involved in protophloem differentiation^43^. The loss-of-function *rpk2* mutant was able partially rescue the protophloem gap phenotypes and root growth of *brx*, *ops* or *cvp2cvl1* mutants^43^. It was shown that *RPK2* interacts with *BAM1* genetically and the two proteins form heteromeric complexes^44^, but the possible interaction of *RPK2* with *BAM3* is not yet explored.

The ability of protophloem sieve element surrounding cells to develop into sieve element cells is important for the developmental plasticity of this tissue^43^. Laser-ablation of developing protophloem triggers the differentiation of the neighboring cells^43^, bypassing the original interrupted protophloem to maintain the supply of sugars and hormones to the meristem. It seems, that the right balance between suppressing and keeping the potential for differentiation to sieve elements in case of failure is essential for the protophloem function. It remains to be answered why maintaining only one single functional sieve element file has such a strict genetic control.

In higher vascular plants, the *CLAVATA* pathway was largely extended both through the number of *CLE* peptide genes, as well as a new components of the receptor complexes, including duplications of *CLV1/BAM*- like RLKs and the appearance of receptor-like protein *CLAVATA2* and pseudo-kinase *CORYNE*^45^. With *CLE33* orthologs found in basal angiosperms, it is possible that during plant evolution, phloem-expressed *CLE*s were recruited to shape the unique angiosperm phloem tissue that functions efficiently in the fast translocation of photoassimilates.

## Methods

### Search of CLE genes in *Arabidopsis thaliana*

A t-BLAST-n search in the *A. thaliana genome* in the *EnsemblPlant* database was conducted using the 32 previously known full-length CLE protein from *A. thaliana* as queries. To get more hits, the *e* value threshold was set to 10 instead of the original setting of 10^−1^. Candidate sequences that were not annotated as *CLE* genes were manually compared to the queries. Only sequences similar to the CLE domain were further analyzed for the presence of a signal peptide, proof of expression, and not being part of another described protein. After filtering, one single sequence in an unannotated region of chromosome 1 remained and possessed all the characteristics of a *CLE* gene: *CLE33*.

### Plant material

All mutants described in this study are in the *Arabidopsis thaliana Columbia-0 (Col-0)* background: *clv2-1*^13^ *crn-10*^13^, *bam1-3* (SALK_015302), *bam1-4* (SALK_107290), *bam2-4* (SAIL_1053_E09), *bam3-2* (SALK_044433)^13^, *bam3-3* (SALK_118860), *bam1-4 bam3-3*, *bam2-4 bam3-2*, *cle45-2*^46^, *brx-3*^47^, *ops-2* (SALK_139316)^8^.

### Generation of CRISPR-Cas9 *cle33*

The loss-of-function mutants in *CLE33* were generated by CRISPR-Cas9 base gene editing. Design of guide-RNAs with low off-targets was performed using CCTop (10.1371/journal.pone.0124633). Generation of the gRNA was achieved by primer dimer annealing (C67xC68; C69xC70) and cloned into the destination vector pAGM55261^48^ by BsaI cut-ligation. *A. thaliana* Col-0 wild-type and *cle45-2* were transformed by a floral dip in *Agrobacterium tumefaciens* suspension. Transgenic seeds were screened for DsRed fluorescence. The frameshift-generating mutations in *CLE33* were searched in the non-transgenic progeny. Higher-order mutants were obtained by crossing.

### Plasmid generation

Genes and promoter regions were amplified by Phusion PCR with the primers indicated in Supplementary Table 1. Plasmids were constructed as indicated in Supplementary Table 2 by Golden Gate modular assembly^49^.

### Phylogenetic and synteny analysis

CLE33/CLE45 and BAM3 homologs were searched by BLASTP in the Phytozome13 and PLAZA Gymnosperm databases. No hints were found in *Carica papaya*, and a t-BLAST-n was performed to find the *CLE33/45* homologs. Tomato CLE sequences were retrieved from^24^. Full-length protein sequences were aligned using either MUSCLE and manually curated in MEGA-X^50^ for Fig. 1a, or MAFFT with DASH enabled^51^ for Supplementary Figs. S9 and S10. The alignments were used to produce trees with 1000 bootstrap replicates with IQTREE^52^, and visualized with iTOL^53^. The synteny analysis of the *CLE33* in the Brassicaceae was performed with the Genome Context Viewer^54^. Multi-sequence alignment profiles were composed with alignmentviewer.org^55^. Sequence conservation logos were generated by WebLogo^56^.

### Root growth assay

Seeds were placed onto 1% sugar half-strength MS plates supplemented or not with the indicated concentration of peptides (Genescript, >75% purity, dissolved in water) or estradiol (stock solution was diluted in DMSO) or corresponding solvent, and kept in dark at 4 °C for 48 h before being transferred in a growth chamber at 22 °C with 16-h-light/8-h-dark cycles. Seedlings were grown for 7 days for root growth inhibition assays by peptides, or 10 days for the genetic suppression of *brx-3/ops-2* root growth phenotypes. After scanning the plates with an Epson scanner at high resolution (600dpi), root length was measured using the single-neurite-tracer tool in Fiji.

### Promoter activity analysis

The promoter region of *CLE33* (876 bp from start codon) was cloned to drive the expression of the GUS reporter system. Transgenic seedlings were incubated at 37 °C in GUS staining buffer (0.5 mg/ml X-Gluc, 100 mM Phosphate buffer, 10 mM EDTA, 1 mM Potassium Ferricyanide, 1 mM Potassium Ferrocyanide, 0.1% Triton X-100) and cleared in 70% ethanol. Images were taken using Zeiss Axioplan microscope and Leica MDG36 stereomicroscope.

For cell-specific expression analysis, upstream regions of *CLE26* (1615bp), *CLE45* (2513 bp), and *CLE33* (876 bp) were cloned and used to drive the expression of nuclear-localized fusion consisting of H2B or NLS and Citrine. For peptide treatment, homozygous transgenic *pCVP2:NLS-3xVenus* seedlings were transferred to the fresh plates containing no peptide, or 100 nM of CLE33p or CLE45p, 22 h before fixation.

### Confocal imaging and sample preparation

Seedlings were fixed with 4% paraformaldehyde (Sigma) in PBS for 1 h minimum. After washing with PBS, samples were stained overnight with a 0.2% Calcofluor White dissolved in a ClearSee solution^57^, and cleared with ClearSee for at least 48 h. Imaging was performed using a Leica TCS SP5 laser-scanning confocal microscope with the following settings: calcofluor-stained cell wall (excitation at 405 nm, detection at 415–500 nm), Venus or Citrine (excitation at 514 nm, detection at 522–574 nm or 524–600 nm).

### Subcellular localization in *Nicotiana benthamiana*

*N. bethamiana* leaves were transiently transformed by infiltration with *A. tumefaciens* carrying a multi-cassette plasmid expressing *pUbi:CLE33-mCherry* and *p35s:Venus*. After 3 days, leaf disks were sequentially imaged with a Leica TCS SP5 laser-scanning confocal microscope for Venus (emission at 514 nm, detection from 524 to 551 nm) and mCherry (emission at 561 nm, detection from 571 to 650 nm).

### qRT-PCR analysis

Five-day-old seedlings were transferred into new plates containing 1 µM Estradiol or the equivalent amount of solvent (DMSO). After 16 hours, seedlings were shock frozen in liquid nitrogen. Frozen samples were grinded in a mill with metal beads. RNA was extracted using the MagMAX Plant RNA Isolation kit (Applied Biosystems). The remaining DNA was eliminated by a 2 M LiCl precipitation. cDNA synthesis was performed using SensiFAST cDNA synthesis kit (Meridian). Quantitative PCRs were performed with Fast Start Universal SYBR-green Master (Roche), with primers from Supplementary Table 1. The thermal cycler (Mic qPCR Cycler, biomolecular systems) conditions were: 95 °C 2 min, 45 cycles of 95 °C 15 s, 58 °C 10 s, 60  °C 50 s, followed by a dissociation curve analysis. Expression values were normalized to *Actin*.

### Statistics and reproducibility

We performed statistical analyses using R v4.0.2 within the Rstudio interface. We used log transformation for root length and distance QC-protophloem cell identity. Statistical significance was determined by ANOVA followed by a post hoc Tukey test for multi-comparison. For gap cell frequency, we employed a *χ*^2^ test with a Benjamini–Hochberg *P* value correction. In general, the sample size was chosen based on the variability observed in preliminary experiments. For assays involving seedlings grown on plates, multiple plates per conditions were used to limit a plate effect variability. We performed root growth assay and root protophloem analysis at least twice with similar results. The expression pattern of CLE33 was derived from more than a dozen of T1 transcriptional reporter lines. Expression analysis by qPCR was performed once with four technical replicates.

### Reporting summary

Further information on research design is available in the Nature Portfolio Reporting Summary linked to this article.

## Supplementary information

Supplementary Material

Reporting summary

## Data Availability

Supplementary datasets supporting this study have been submitted to Dryad and can be found using this link: 10.5061/dryad.x69p8cznw. These datasets include the numerical source data for graphs and charts that can be found in the Excel file, original confocal images in *lif* and *lsm* formats, and gene alignments in *fas* format. All additional data are available from the corresponding author upon reasonable request.
