# Supplementary material for: The CLE33 peptide represses phloem differentiation via autocrine and paracrine signaling in Arabidopsis
Source: Commun Biol. 2023 Jun 6;6:588. doi: 10.1038/s42003-023-04972-2 (PMC10244433; doi:10.1038/s42003-023-04972-2)
Supplement: Supplementary file 1 — Supplementary Material [file 42003_2023_4972_MOESM1_ESM.pdf]

The CLE33 peptide represses phloem differentiation *via* autocrine and paracrine signaling in Arabidopsis

Samy Carbonnel[1], Salves Cornelis[1] and Ora Hazak[1*]

1- Department of Biology, University of Fribourg, Chemin du Musee 10, 1700 Fribourg, Switzerland

*- corresponding author ora.hazak@unifr.ch

Supplementary Figures (1-10) and Tables (1-2).

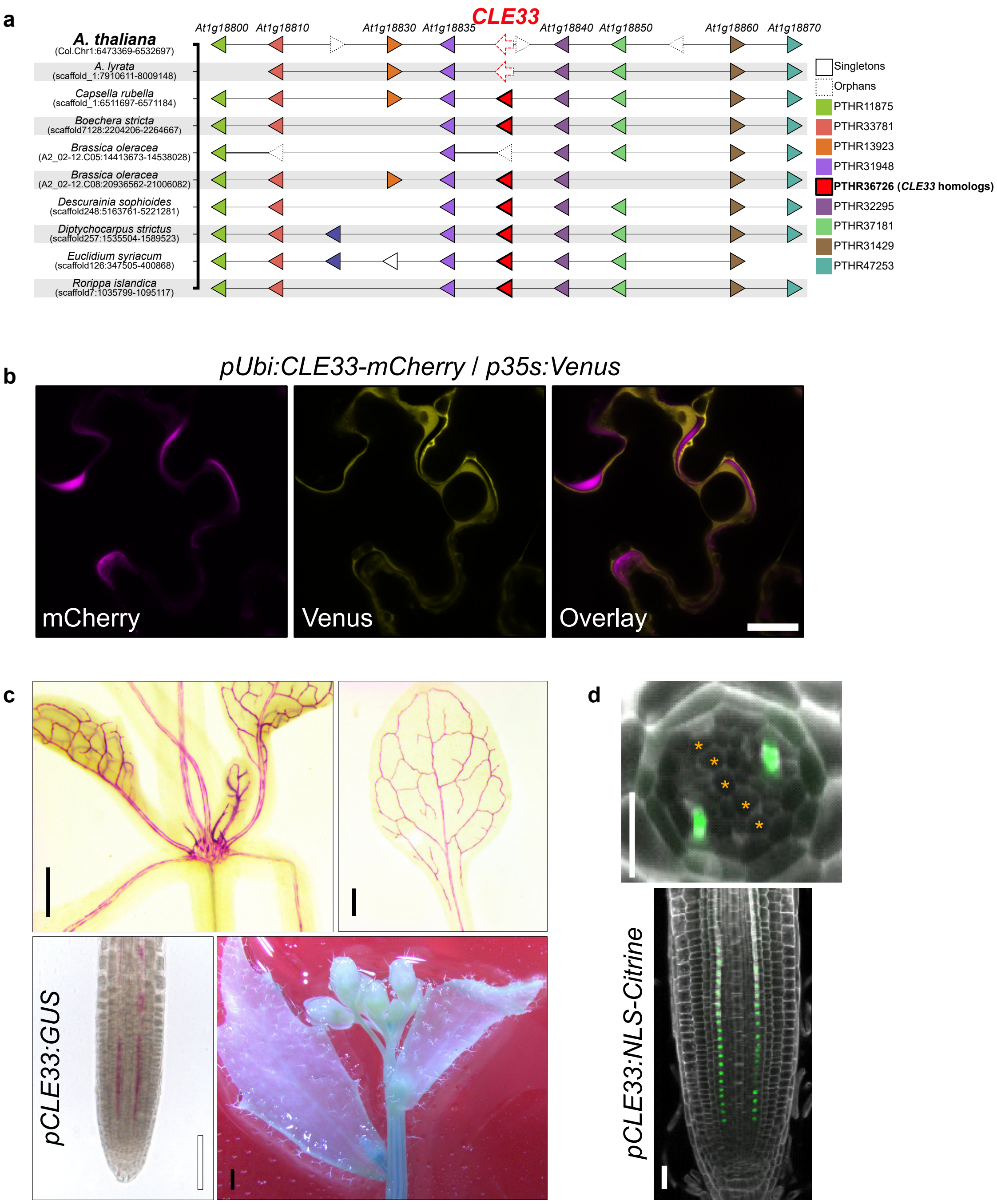

Supplementary Figure 1. a. Syntenic context of the *CLE33* locus on chromosome 1 in other Brassicaceae species. In *Arabidopsis thaliana* and *lyrata CLE33* genes position and orientation are represented by a doted red arrow. The family ID PTHR36726 refers to *CLE33* orthologs. **b.** Confocal images of *CLE33-mCherry* transiently expressed in *Nicotiana benthamiana* leaves, showing apoplast localization. Scale bar corresponds to 25 µm. **c.** Vascular *CLE33* expression revealed by transcriptional-GUS fusion in rosette junction (upper-left), cotyledon (upper-right), root apex (lower-left), inflorescence (lower-right). The black scale bars correspond to 1 mm and white scale bar corresponds to 100 µm **d.** *CLE33* is expressed in developing protophloem sieve element cells. Orange asterisks indicate xylem cells. The scale bar in the upper image (optical cross-section) corresponds to 25 µm and in the lower (longitudinal section) image 50 µm.

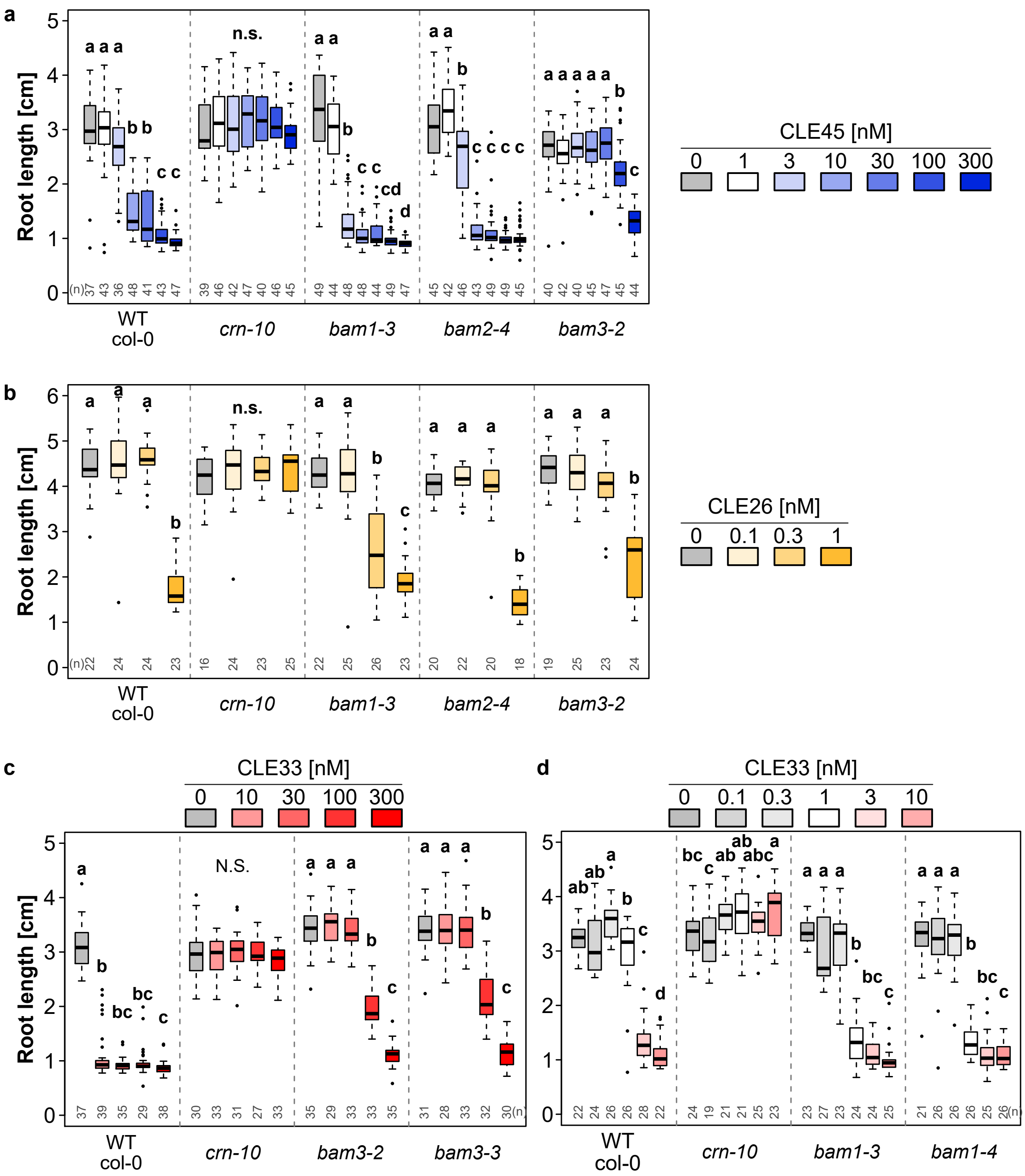

Supplementary Figure 2. CLE33/CLE45 *BAM3*-dependent root growth arrest. a-d. Dose dependent root growth response to synthetic CLE45 (**a**), CLE26 (**b**) and CLE33 peptides (**c-d**). Letters indicate different statistical group (ANOVA, post-hoc Tukey test).

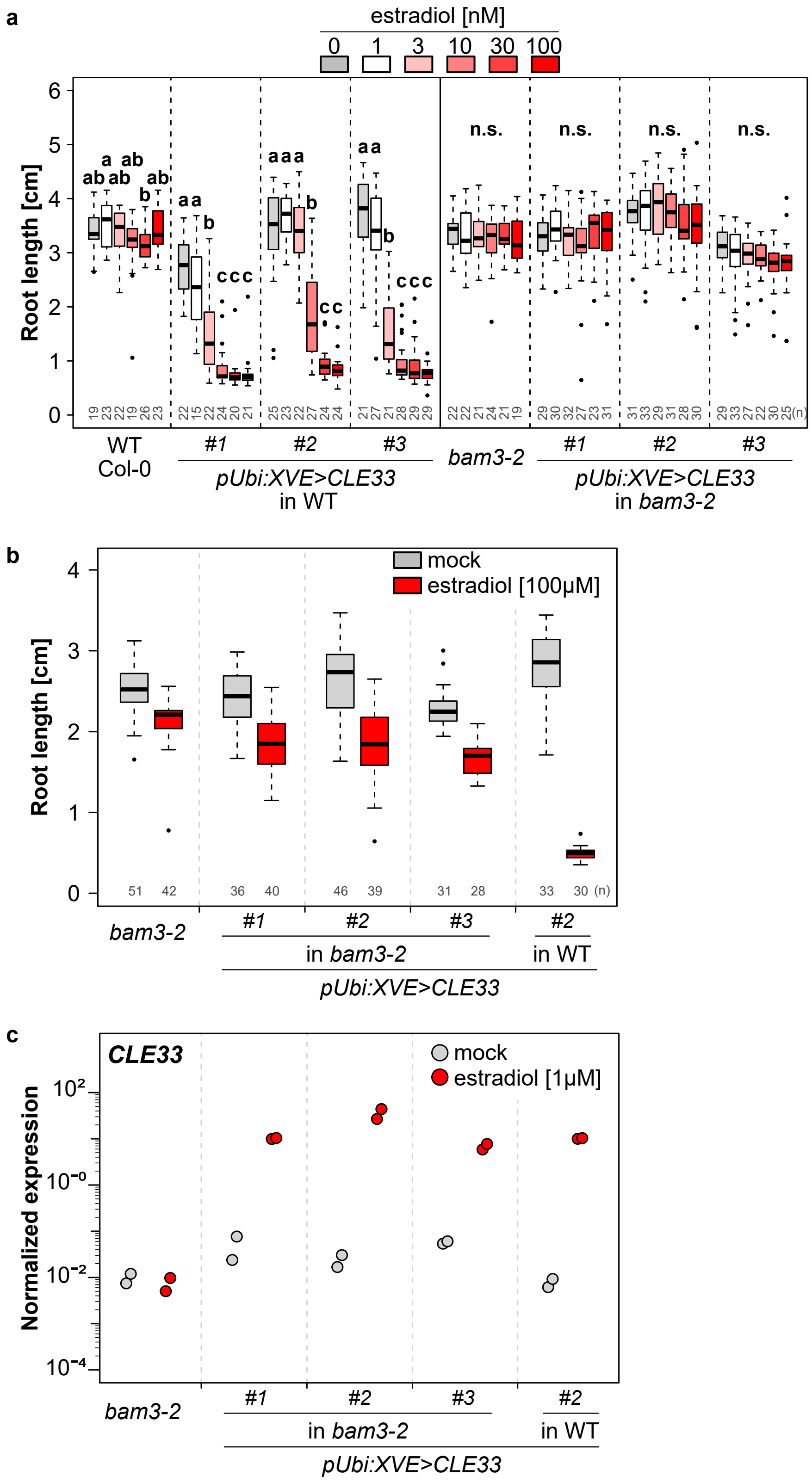

Supplementary Figure 3. Dose-dependent root growth arrest by inducible over-expression of *CLE33* requires *BAM3*. a-b. Dose dependent root growth response to estradiol-inducible lines expressing *CLE33* in wild-type and *bam3-2* mutant. Letters show different statistical group (ANOVA, post-hoc Tukey test). **c.** Transcriptional induction of *CLE33* by overnight estradiol treatment detected by qPCR (n=2).

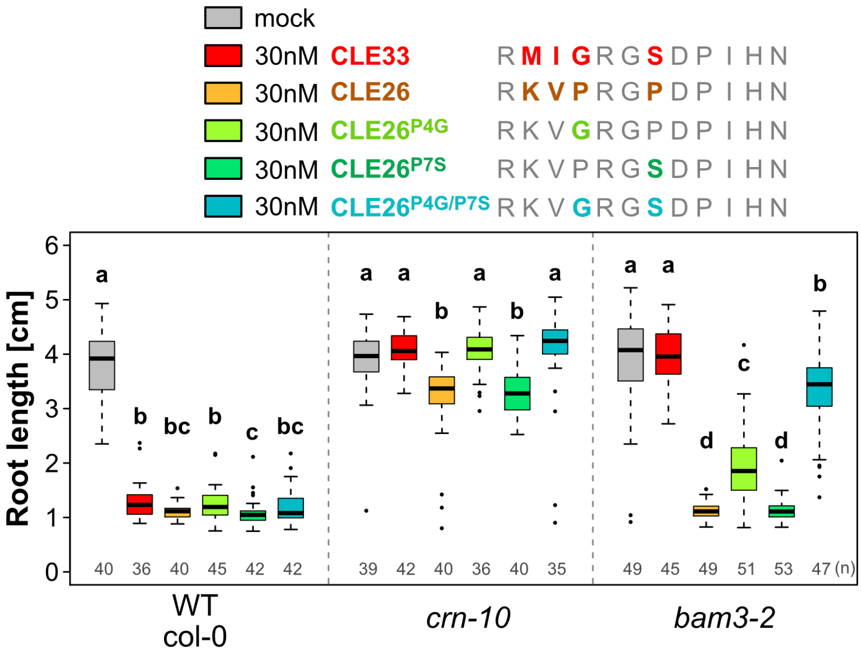

Supplementary Figure 4. Substitution of proline residues in CLE26 in positions 4 and 7 to glycine and serine leads to *BAM3*-dependent root responses. Letters indicate different statistical group (ANOVA, post-hoc Tukey test).

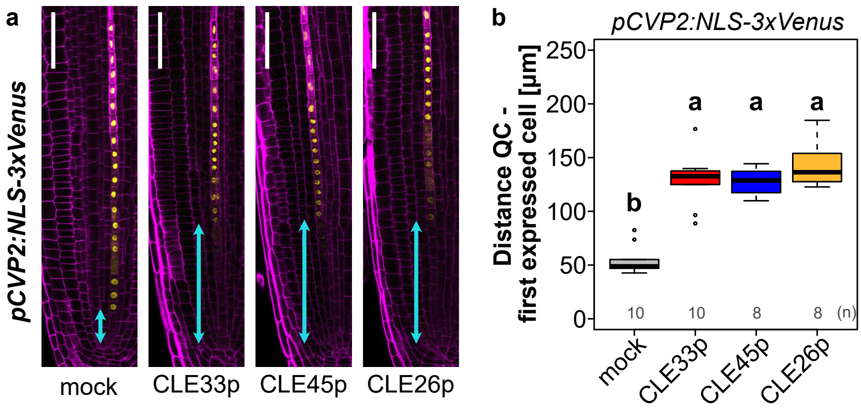

Supplementary Figure 5. CLE33 inhibits protophloem development. a. Confocal images of the protophloem cell identity marker *pCVP2:NLS-3xVenus* upon treatment with phloem peptides. Cyan arrows indicate the distance between the first fluorescent cells and the quiescent centre, quantified in **b**. Letters indicate different statistical group (ANOVA, post-hoc Tukey test). Scale bars correspond to 50 µm.

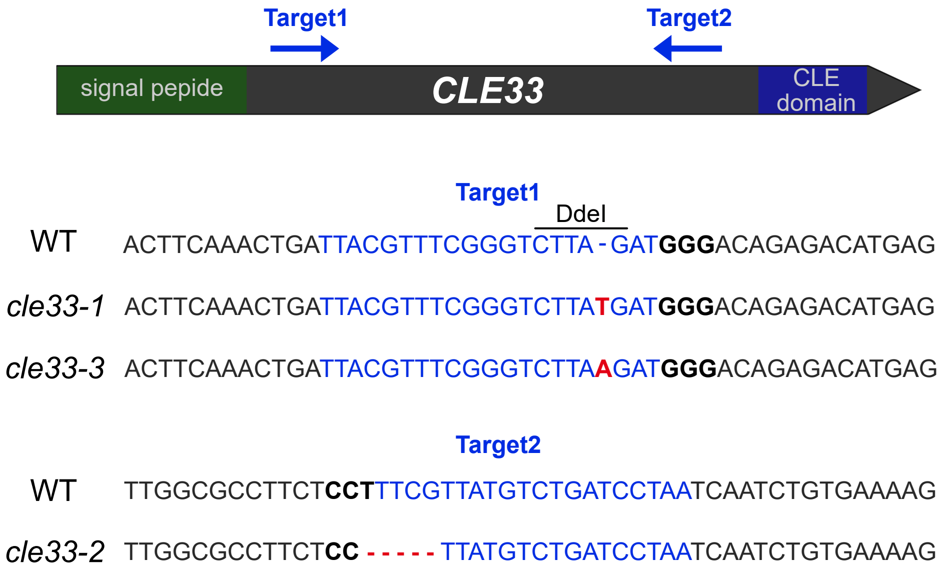

Supplementary Figure 6. CRISPR-Cas9 targeted regions in *CLE33*. Blue arrows indicate the single-guide RNAs. Protospacer-adjacent motifs (PAM) are in bold. Indels are indicated in bold red for each mutant. The endonuclease DdeI recognition site is indicated by a black line.

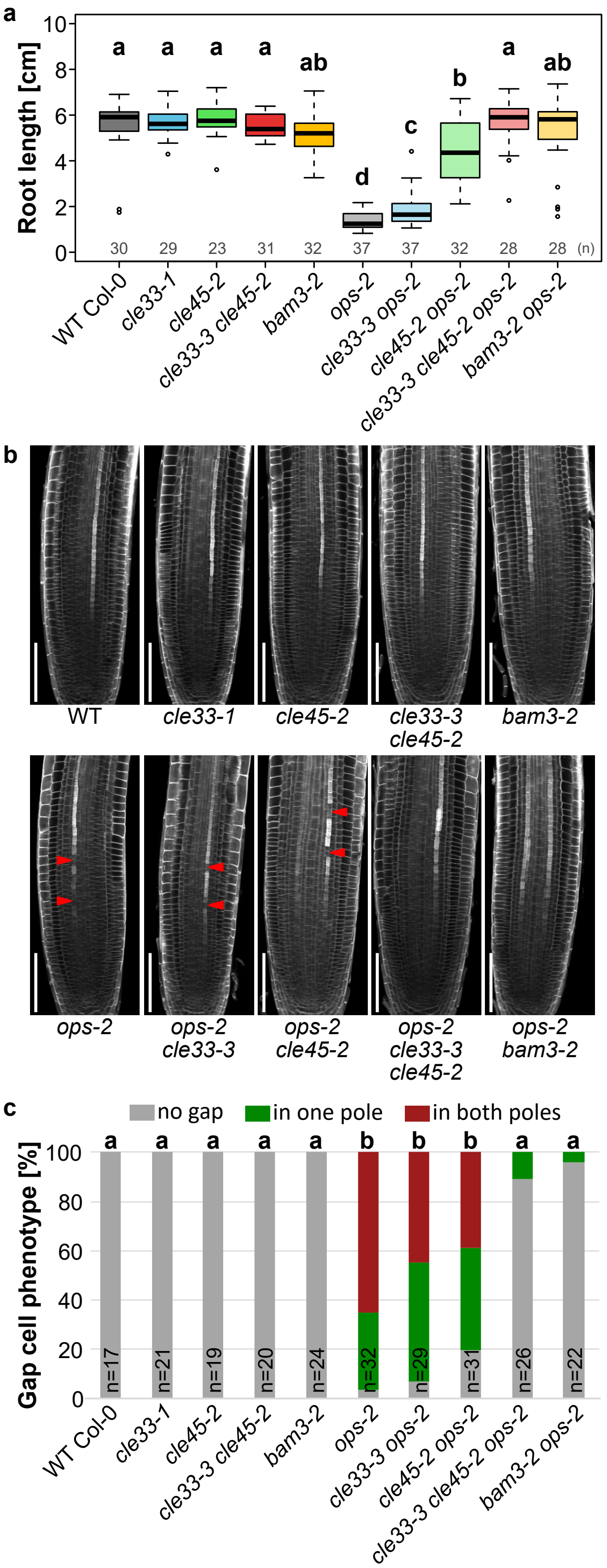

Supplementary Figure 7. *CLE33* and *CLE45* act in concert in *OPS*-mediated protophloem development. a. Seedling root length at 10 days post germination. Letters indicate different statistical group (ANOVA, post-hoc Tukey test). **b.** Representative confocal images of calcofluor white stained roots presenting protophloem differentiation continuity. Red arrows indicate protophloem gap cells. Scale bars correspond to 100 µm. **c.** Quantification of the gap cell phenotype frequency. Letters indicate different statistical group (χ^2 test with Benjamini-Hochberg correction).

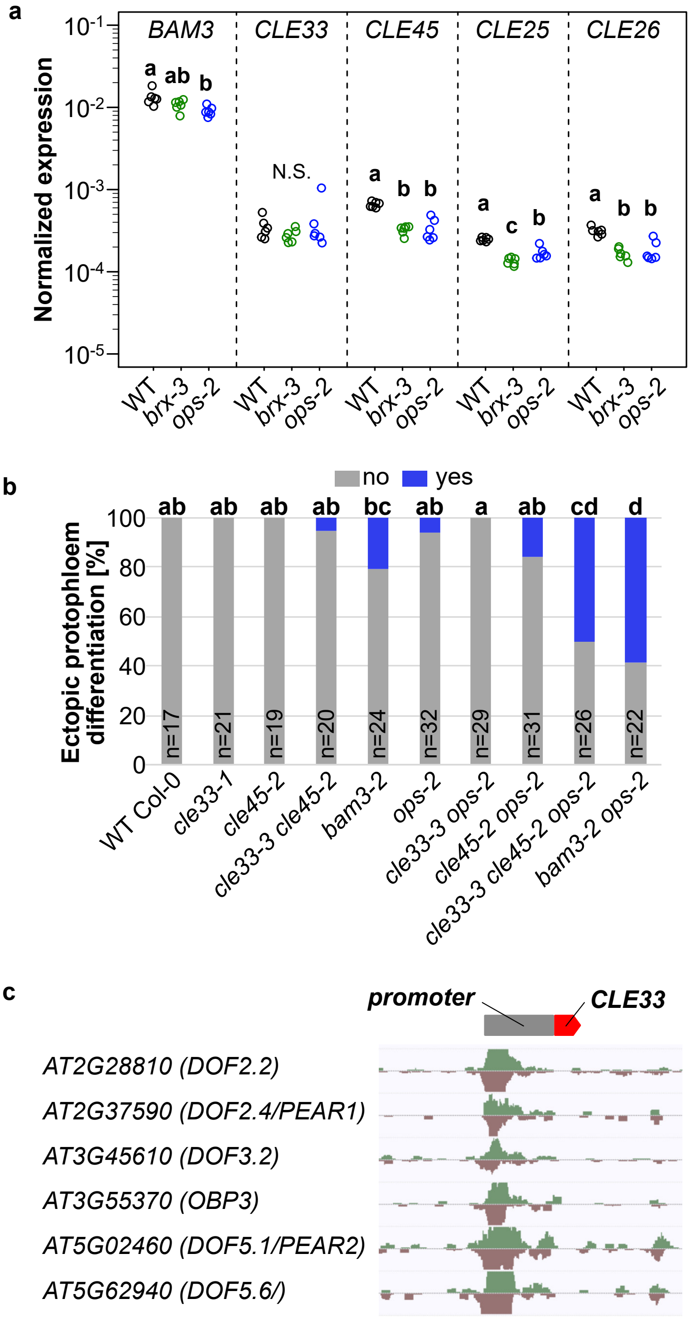

Supplementary Figure 8. CLE33/45-BAM3 module represses ectopic protophloem differentiation. a. Expression levels of *BAM3* and phloem-specific *CLE*s in the root tissues of *brx-3* and *ops-2* mutants tested by q-PCR (n=6). Letters indicate different statistical group (χ^2 test with Benjamini-Hochberg correction). **b.** Percentage of roots with ectopic sieve element-like cells. **c.** DAP-seq peaks (in-vitro binding sites) of selected DOFs transcription factors (Plant Cistrome database, http://neomorph.salk.edu). Cloned promoter for transcriptional fusions and *CLE33* coding sequence are indicated in grey and red, respectively.

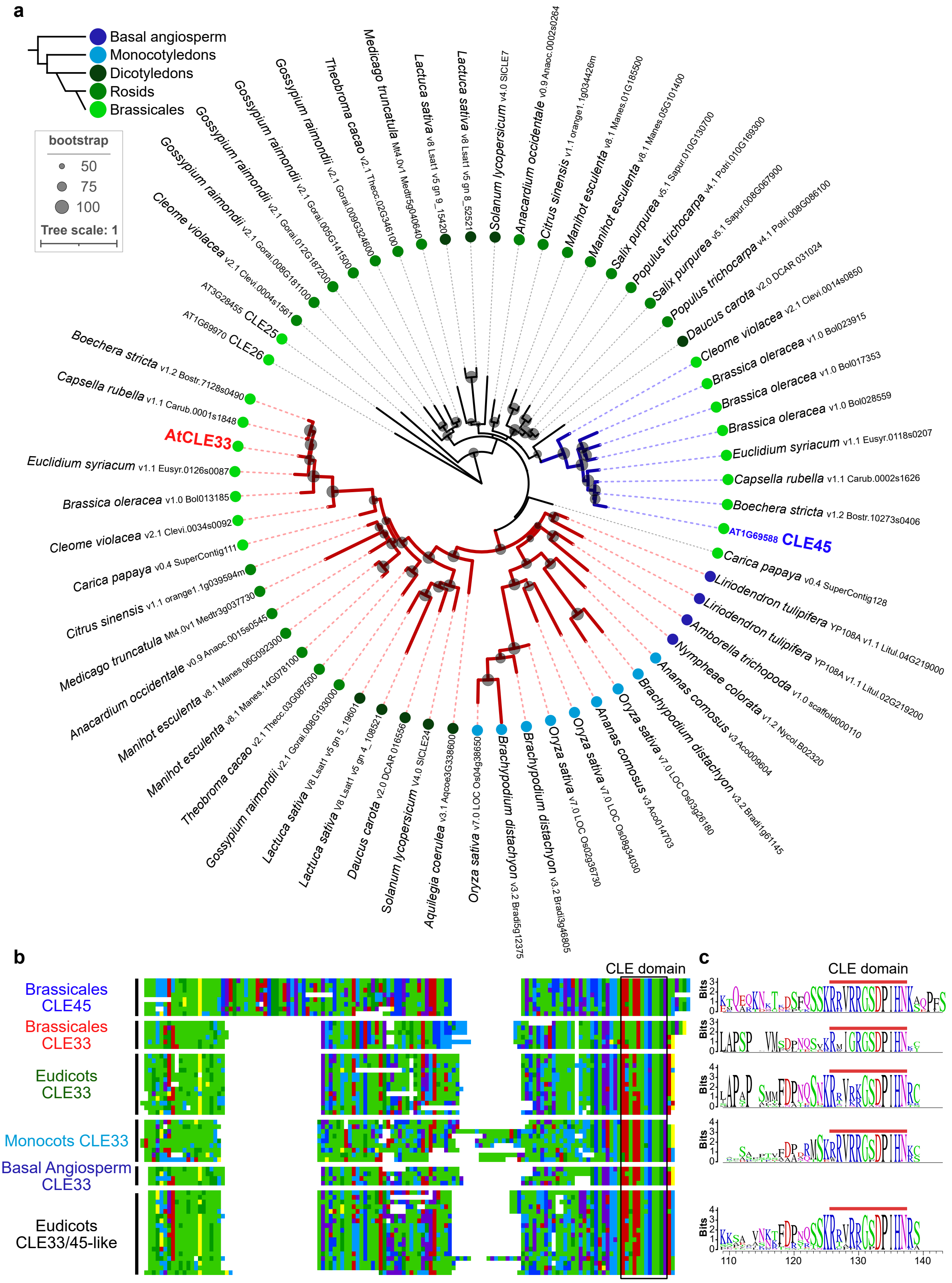

Supplementary Figure 9. *CLE33* orthologs are present in basal angiosperms. a. Phylogenetic tree of the *CLE33* and *CLE45* orthologs (shown in red and blue branches, respectively). The tree is rooted with *AtCLE25* and *AtCLE26*. **b.** Multi sequence alignment of *CLE33/45* homolog sequences deriving out of the phylogenetic tree shown in **a**. Group of eudicots *CLE33/45*-like genes corresponds to the other sequences not being identified as

CLE33 or *CLE45* orthologs. **c.** Conservation LOGO of the CLE propeptide highlighting the C-terminal part according to their evolutionary group shown in **b**.

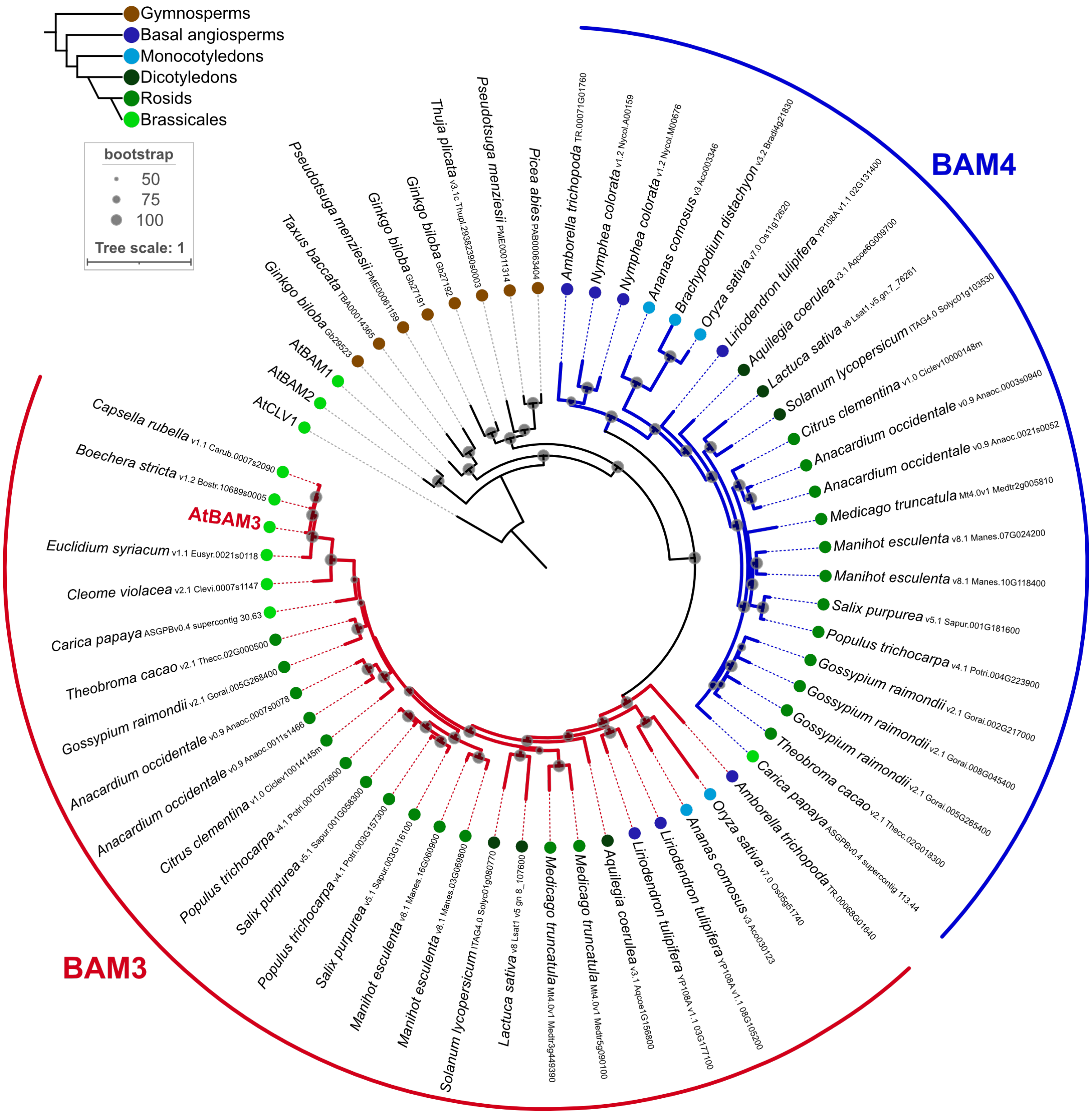

Supplementary Figure 10. Phylogenetic tree of the *BAM3* and *BAM4* orthologs. Remarkable absence of *BAM4* in *Brassicaceae*, a copy that emerged from a duplication of *BAM3* in early angiosperms. Tree is rooted with *AtCLV1*.

Supplementary Table 1. Primers used in this study.

**Cloning**		
LI Esp3I A-B *pAtCLE33*	C62	TATCGTCTCAGCGGGTATAATGAACTAATTATCCTTAAGCTCCTCATATGT
	C63	TATCGTCTCACAGAGTTAACTAATATGTAGATACTACAGCGTTTTGTTTCT GAAAG
LI Esp3I A-B *pAtCLE45*	E42	TATCGTCTCAGCGGTTGATTAATCAAGAATGCACAATTG
	E43	TATCGTCTCACAGATTCTGCTCTTAGGCAGACAAG
LI Esp3I A-B *pAtCLE26*	E40	TATCGTCTCAGCGGCGAGCAAATTTTCTCGTTGG
	E41	TATCGTCTCACAGAGGTTTCTAGCCTTTGTGGATA
LI Esp3I C-D *AtCLE33*	H33	TATCGTCTCACACCATGGGTGTTTCAATTGGTCATATAGGAATGA
	H34	TATCGTCTCACCTTTCAGCATTTGTTGTATTTGTTGTGAATAGGAT
LI BsaI C-D *AtCLE33 noStop*	C64	TATGGTCTCACACCATGGGTGTTTCAATTGGTCATATAGGAATGATTC
	C65	TATGGTCTCACCTTGCATTTGTTGTATTTGTTGTGAATAGGATCAGATC
LI BsaI C-D *AtCLE33*	C64	TATGGTCTCACACCATGGGTGTTTCAATTGGTCATATAGGAATGATTC
	C66	TATGGTCTCACCTTTCAGCATTTGTTGTATTTGTTGTGAATAGGATCAG
LI B-C *NLS_H2B*	C60	TATGGTCTCATCTGATGGCGAAGGCAGATAAGAAA
	C61	TATGGTCTCAGGTGAGAGAACTCGTAAACTTCGTAACC
LI C-D *Citrine*	C58	TATGGTCTCACACCATGGTGAGCAAGGGCGAGG
	C59	TATGGTCTCACCTTTTACTTGTACAGCTCGTCCATGCC
LI A-G FastRed	PP494	ATGAAGACTTTACGGGTCTCAGCGGAATGTCGCGGAACAAATTTTAAAAC
	PP495	TAGAAGACAATCGAGAAGTCACGTGTCAATG
	PP496	ATGAAGACTTTCGACTCCTTTCTTAATATATCTAACAAAC
	PP497	ATGAAGACTTCAGAGGTCTCAGACAACTAAATGGAGCAACCTACTG
LI B-C *XVE*	G58	ATGAAGACTTTACGGGTCTCATCTGATGAAAGCGTTAACGGCCAGG
	G59	GAAGACTGGACTCGTCTGGCGCTCCATG
	G60	GAAGACCGAGTCCAATCATCAGGATCTCTAG
	G61	GAAGACAGGAAACGGACCAAAGCCACTTG
	G62	GAAGACCGTTTCCTCCACGGATGCCC
	G63	ATGAAGACTTCAGAGGTCTCAGGTGTGCTAGAGTCGACTAGCTTCAGCG

**Guide RNAs**		
gRNA1	C67	ATTGTTACGTTTCGGGTCTTAGAT
	C68	AAACATCTAAGACCCGAAACGTAA
gRNA2	C69	ATTGTTAGGATCAGACATAACGAA
	C70	AAACTTCGTTATGTCTGATCCTAA

**qPCR**		
AtCLE33	J77	ACGTTTCGGGTCTTAGATGGG
	J78	AGGCGCCAAGTTTCCTTTTG
AtActin	E08	CCGATCCAGACACTGTACTTCCTT
	E09	CTTGCACCAAGCAGCATGAA

New mutants genotyping			
cle33-1 & *cle33-3*	C64	TATGGTCTCACACCATGGGTGTTTCAATTGGTCATATAGGAATGATTC	DdeI (cut WT)
	J11	GAACACGCAAAGGATGAACA	
cle33-2	H31	TGGGACAGAGACATGAGGATGCA	Sequencing
	H34	TATCGTCTCACCTTTCAGCATTTGTTGTATTTGTTGTGAATAGGAT	
bam3-3	J16	TCCCGAGGTGAAATCTCCGCAA	WT
	J17	TGAACAAAAAGCGAGCAATCTCA	
	J16	TCCCGAGGTGAAATCTCCGCAA	T-DNA insertion
	E17	ATTTTGCCGATTTCGGAAC	

Supplementary Table 2. Cloning steps.

Name	Description
**Golden Gate Level 0**	
L0 B-C XVE A	PCR amplification from pMDC7 with G58+G59 primers. Assembled by blunt ligation into BB01
L0 B-C XVE B	PCR amplification from pMDC7 with G60+G61 primers. Assembled by blunt ligation into BB01
L0 B-C XVE C	PCR amplification from pMDC7 with G62+G63 primers. Assembled by blunt ligation into BB01
**Golden Gate Level I**	
LI B-C XVE	Assembled by BpiI cut-ligation from: L0 B-C XVE A + L0 B-C XVE B + L0 B-C XVE C, into BB03
LI Esp3I A-B *pAtCLE33*	PCR amplification of *A. thaliana* genomic DNA with C62+C63 primers. Assembled by blunt ligation into BB02
LI Esp3I A-B *pAtCLE45*	PCR amplification of *A. thaliana* genomic DNA with E42+E43 primers. Assembled by blunt ligation into BB02
LI Esp3I A-B *pAtCLE26*	PCR amplification of *A. thaliana* genomic DNA with E40+E41 primers. Assembled by blunt ligation into BB02
LI Esp3I C-D *gAtCLE33*	PCR amplification of *A. thaliana* genomic DNA with H33+H34 primers. Assembled by blunt ligation into BB02
LI BsaI C-D *gAtCLE33*	PCR amplification of *A. thaliana* genomic DNA with C64+C66 primers. Assembled by blunt ligation into BB02
LI BsaI C-D *gAtCLE33* noSTOP	PCR amplification of *A. thaliana* genomic DNA with C64+C65 primers. Assembled by blunt ligation into BB02
LI B-C *NLS_H2B*	PCR amplification from pENTRY H2B with C60+C61 primers. Assembled by blunt ligation into BB01
LI C-D *Citrine*	PCR amplification from pENTRY Citrine with C58+C59 primers. Assembled by blunt ligation into BB02
LI A-G FastRed	PCR amplifications from pAGM55261 with PP494+PP495 and PP496+PP497. Assembled by BpiI cut ligation into BB03
**Golden Gate Level II**	
LII F1-2 FastRed	Assembled by BsaI cut-ligation from: LI A-G FastRed into LIIc F1-2 (BB30)
LII F3-4 *pOI:Gus*	Assembled by BsaI cut-ligation from: LI A-B pOI (G082) + LI B-E Gus (G79) + LI E-F HSP-term (G45) + LI F-G dy (BB09) into LIIc F3-4 (BB33)

LII F3-4 *pOI:H2B-Citrine*	Assembled by BsaI cut-ligation from: LI A-B pOI (G082) + LI B-C NLS_H2B + LI C-D Citrine + LI D-E dy (BB08) + LI E-F NOS-term (G06) + LI F-G dy (BB09) into LIIc F3-4 (BB33)
LII F3-4 *pOI:XVE>GOI*	Assembled by BsaI cut-ligation from: LI A-B pOI (G082) + LI B-C XVE + LI C-D GOI (G83)+ LI D-E dy (BB08) + LI E-F HSP-term (G45) + LI F-G dy (BB09) into LIIc F3-4 (BB33)
LII F3-4 *pUbi:CLE33-mCherry*	Assembled by BsaI cut-ligation from: LI A-B pAtUbi + LI B-C dy (BB06) + LI C-D gAtCLE33 noStop + LI D-E mCherry (G25) + LI E-F HSP-term (G45) + LI F-G dy (BB09) into LIIc F3-4 (BB33)
LII F5-6 *p35s:Venus*	Assembled by BsaI cut-ligation from: LI A-B p35s (G05) + LI B-C dy (BB06) + LI C-D Venus (G20) + LI D-E dy (BB08) + LI E-F NOS-term (G06) + LI F-G dy (BB09) into LIIc F5-6 (BB36)
**Golden Gate Level III**	
LIII FastRed / *pOI:Gus*	Assembled by BpiI cut-ligation from: LII F1-2 FastRed + LII 2-3 ins (BB44) + LII F3-4 *pOI:Gus* + LII 4-6 dy (BB41) into LIIIβ Fin (BB52)
LIII FastRed / *pCLE33:Gus*	Assembled by Esp3I cut-ligation from: LI A-B *pAtCLE33* into LIII FastRed / pOI:Gus
LIII FastRed / *pOI:H2B-Citrine*	Assembled by BpiI cut-ligation from: LII F1-2 FastRed + LII 2-3 ins (BB44) + LII F3-4 *pOI:H2B-Citrine* + LII 4-6 dy (BB41) into LIIIβ Fin (BB52)
LIII FastRed / *pCLE33:H2B-Citrine*	Assembled by Esp3I cut-ligation from: LI A-B Esp3I *pAtCLE33* into LIII FastRed / *pOI:H2B-Citrine*
LIII FastRed / *pCLE45:H2B-Citrine*	Assembled by Esp3I cut-ligation from: LI A-B Esp3I *pAtCLE45* into LIII FastRed / *pOI:H2B-Citrine*
LIII FastRed / *pCLE26:H2B-Citrine*	Assembled by Esp3I cut-ligation from: LI A-B Esp3I *pAtCLE26* into LIII FastRed / *pOI:H2B-Citrine*
LIII *pAtUbi:CLE33-mCherry / p35s:Venus*	Assembled by BpiI cut-ligation from: LII 1-3 dy (BB38) + LII F3-4 *pUbi:CLE33-mCherry* + LII 4-5 ins (BB43) + LII F5-6 *p35s:Venus* into LIIIβ Fin (BB52)
LIII FastRed / *pOI:XVE>GOI (LacZ)*	Assembled by BpiI cut-ligation from: LII F1-2 FastRed + LII 2-3 ins (BB44) + LII F3-4 *pOI:XVE>GOI* + LII 4-6 dy (BB41) into LIIIβ Fin (BB52)
LIII FastRed / *pOI:XVE>GOI (ccdb)*	Assembled by Esp3I cut-ligation from: LI A-B pOI (G84) + LI C-D GOI (G85) into LIII FastRed / *pOI:XVE>GOI*(LacZ)
LIII FastRed / *pUbi:XVE>CLE33*	Assembled by BsaI cut-ligation from: LI A-B pAtUbi + LI BsaI C-D *gAtCLE33* into LIII FastRed / *pOI:XVE>GOI*(LacZ)
pAGM55261_AtCLE33gRNA1	Assembled by BsaI cut-ligation from primer dimer of gRNA1 into pAGM55261
pAGM55261_AtCLE33gRNA2	Assembled by BsaI cut-ligation from primer dimer of gRNA2 into pAGM55261
